# A mechanistic approach for accurate simulation of village scale malaria transmission

**DOI:** 10.1186/1475-2875-8-223

**Published:** 2009-10-02

**Authors:** Arne Bomblies, Jean-Bernard Duchemin, Elfatih AB Eltahir

**Affiliations:** 1 Massachusetts Institute of Technology, Cambridge, MA 02139, USA; 2 Institute Pasteur, Paris, France; 3 221 Votey Hall, 33 Colchester Ave, University of Vermont, Burlington, VT 05405, USA

## Abstract

**Background:**

Malaria transmission models commonly incorporate spatial environmental and climate variability for making regional predictions of disease risk. However, a mismatch of these models' typical spatial resolutions and the characteristic scale of malaria vector population dynamics may confound disease risk predictions in areas of high spatial hydrological variability such as the Sahel region of Africa.

**Methods:**

Field observations spanning two years from two Niger villages are compared. The two villages are separated by only 30 km but exhibit a ten-fold difference in anopheles mosquito density. These two villages would be covered by a single grid cell in many malaria models, yet their entomological activity differs greatly. Environmental conditions and associated entomological activity are simulated at high spatial- and temporal resolution using a mechanistic approach that couples a distributed hydrology scheme and an entomological model. Model results are compared to regular field observations of *Anopheles gambiae sensu lato *mosquito populations and local hydrology. The model resolves the formation and persistence of individual pools that facilitate mosquito breeding and predicts spatio-temporal mosquito population variability at high resolution using an agent-based modeling approach.

**Results:**

Observations of soil moisture, pool size, and pool persistence are reproduced by the model. The resulting breeding of mosquitoes in the simulated pools yields time-integrated seasonal mosquito population dynamics that closely follow observations from captured mosquito abundance. Interannual difference in mosquito abundance is simulated, and the inter-village difference in mosquito population is reproduced for two years of observations. These modeling results emulate the known focal nature of malaria in Niger Sahel villages.

**Conclusion:**

Hydrological variability must be represented at high spatial and temporal resolution to achieve accurate predictive ability of malaria risk at the village scale, which can then be integrated appropriately to regional spatial scales and seasonal temporal scales. These results have important implications for models seeking to link the impacts of climate change and climate variability to malaria transmission. The highly focal nature of malaria in the Sahel makes detailed representation necessary to evaluate village-level risks associated with hydrology-related vector population variability.

## Background

In the Niger Sahel, small-scale, ephemeral pools dot the landscape following monsoon rainstorms and facilitate the prolific *Anopheles gambiae sensu lato *mosquito breeding that brings about a pronounced seasonal malaria increase [1]. The sudden appearance of widespread but temporary water pools gives rise to a rapid increase in mosquito populations clustered around human habitation, resulting in the highly focal malaria transmission that is a characteristic of Sahel villages [1,2]. Inter-village differences in local hydrology can lead to unequal extents of suitable breeding habitat following rain, and consequently different villages exhibit different levels of transmission. Village-scale hydrological conditions therefore become important determinants of local malaria transmission [1,2].

Water-limited savanna environments such as the Sahel exhibit spatial variability in hydrology associated with the local dominance of either trees or grasses. This is largely due to topographic effects and applies to a region of climatic conditions spanned by the very dry conditions in the north and the uniformly wet conditions in the south [3]. In regions south of the Sahel, wet conditions prevail and water availability does not limit mosquito populations. North of the Sahel it is too dry for mosquitoes to breed consistently. Both of these bounds of the Sahel exhibit low spatial hydrological variability. Because spatial variability of malaria transmission is associated with the local-scale availability of surface water for mosquito breeding, the Sahel represents a relative maximum in spatial variability of hydrology [3] as well as malaria transmission.

Despite the heterogeneous, focal malaria transmission resulting from spatial variability in environmental conditions [2], computer models seeking to predict the impacts of climate variability on malaria transmission dynamics typically lump environmental variables such as temperature and some measure of habitat suitability in large computational grid cells of tens to hundreds of kilometers in size, smoothing over any potential heterogeneity within these areas [4]. The coarse resolution effectively negates the potential effects of strong--but localized--proximal determinants of malaria for specific villages. Here, the importance of this lost sub-grid cell heterogeneity in habitat-forming conditions is highlighted by simulating the impacts of small-scale spatial hydrological variability on village-scale malaria transmission. Field hydrology and entomology data are presented from two villages in southwestern Niger, located only 30 km apart--a much smaller distance than a typical climate model grid cell--but with dramatically different hydrological and mosquito population characteristics. At these two villages, we simulate the village-scale anopheles mosquito population dynamics using a high resolution, distributed, coupled hydrology and entomology model HYDREMATS [1].

The characteristic spatial scale of anopheles mosquito population dispersal surrounding African villages is approximately 1-2 kilometers [2,5-7], with the most dangerous breeding habitats often occurring within tens to hundreds of meters of the nearest human habitation [1,8]. Some references list much longer mosquito flight ranges up to 10 km [e.g. [2,9]] but the bulk of entomological activity directly affecting Sahel villages is concentrated in an area surrounding villages spanning one or two kilometers [6,7]. Building on previous work [1], this study aims to represent the impacts of hydrological variability at the village scale and demonstrate through computer model simulations the importance of high resolution hydrological variability for the prediction of village-scale malaria transmission.

## Methods

### Field observations

**Figure 1:**
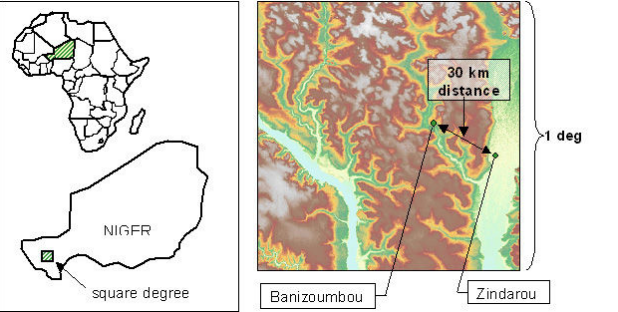
**Locations of the study villages Banizoumbou and Zindarou, Niger**. The left panel shows the location of the square degree zone (2-3°E, 13-14°N) depicted in the right panel, which depicts topographic relief. The major fluvial feature in the right of the zone is the relict river valley known as the "Dallol Bosso".

Banizoumbou and Zindarou, Niger, are separated by only 30 km, however they represent two drastically different hydrological environments. See Figure 1 for the locations of Banizoumbou and Zindarou. Banizoumbou is typical of the Niger Sahel: dry savannah conditions prevail and vegetation is dominated by wet-season millet crops and tiger bush shrubland. Soil is predominantly sand (about 90%), and natural water bodies are exclusively rainfed pools that rapidly lose water to evaporation and infiltration. The groundwater table is about 25 meters below the surface. In contrast, Zindarou has a much wetter environment due to the presence of a shallow groundwater table which is approximately one meter below the ground surface at the peak of the monsoon. As the monsoon progresses, the rising groundwater table penetrates the land surface in topographic low points, creating large water bodies (~100 m) that persist for many weeks, and recede after the cessation of rains in September. The shallow groundwater is the legacy of a major river system draining what is now the central Sahara [10]. Zindarou is situated in the floodplain of this abandoned river, which is known as the "Dallol Bosso". Relict braided channel features are identifiable in satellite images, and these features typically constitute the low points allowing the surface expression of groundwater. Extensive and prolonged mosquito breeding results from this hydrological phenomenon. Consequently, Zindarou is plagued with many more Anopheles mosquitoes than other nearby villages during the wet season.

Mosquitoes were collected at Banizoumbou and Zindarou weekly during the 2005 and 2006 wet seasons, and monthly during the dry seasons. During each field visit, CDC light traps were placed at six locations in each village. Each trap was powered with a freshly charged motorcycle battery, and was started at 7 PM and stopped at 7 AM the following morning. Mosquitoes were separated from the catch and then identified to species using a microscope. In each village, three of the traps were placed inside three houses near the inhabitants' beds, and three others were placed outside of the same three houses. Bednets were not used by the inhabitants. The sampled houses were constructed of mud brick walls with a thatch roof, or completely of thatch. Light trap locations were chosen in order to provide a representative sample of entomological activity in the village by sampling a variety of housing types and a variety of locations relative to breeding habitat. All sampled houses have open eaves and open doorways.

Meteorological stations were operating in both Zindarou and Banizoumbou for the duration of the study. Temperature, humidity, incident solar radiation, and wind speed and direction were sampled every 15 minutes in both villages, and recorded in dataloggers (Campbell Scientific CR10X). In addition, soil moisture was monitored at three sampling sites in each village, with four Campbell Scientific time domain reflectometry (TDR) probes at each station sampling volumetric water content in a vertical profile configuration at 10 cm, 20 cm, 50 cm and 100 cm below the ground surface. These soil moisture values inform unsaturated zone hydrology and provide a calibration target for simulated soil moisture. In 2005, 405 mm of rain fell in Banizoumbou, while Zindarou saw 348 mm of rainfall excluding much of June. The meteorological station in Zindarou was installed on June 23, 2005, and missed three weeks of the June precipitation. June rainfall was approximated using Banizoumbou June precipitation data, which was 66.5 mm (16% of total) until June 23. In 2006, the Banizoumbou rain gauge registered 478 mm while the Zindarou precipitation totalled 460 mm. Average rainy season temperatures in each village (June-September) were 28.6°C in 2005 in Banizoumbou, and 29.0°C in Zindarou. For 2006, the rainy season temperatures were 29.3°C and 29.4°C in Banizoumbou and Zindarou, respectively.

All surface water bodies within one kilometer of each village were monitored weekly for anopheles mosquito larval activity using a standard dipper. Ten dips were taken at each location during each field visit. For each dip, anopheles larvae of each instar (L1-L4) and pupae were counted. Because Banizoumbou light trap captures included only *An. gambiae s.l*., we assumed that all anopheles subadults seen in Banizoumbou pools are *An. gambiae s.l*. Various types of aquatic habitat are present in Zindarou, including permanent shallow garden wells, long-duration (multi-week) surface expressions of groundwater as well as short-duration (multi-day) rainfed puddles similar to those found in Banizoumbou. All of these Zindarou water bodies regularly contained *An. gambiae *larvae in addition to other anophelines. Larval samples were taken and reared to adulthood. These were used to confirm various habitat usage by *An. gambiae*. Because virtually no sizeable water bodies in Zindarou were consistently devoid of larvae, and because larval surveys are generally not a good measure of absolute abundance, little emphasis is placed on the numbers from the larval counts. Instead, the larval counts are valuable as qualitative indicators of pool productivity and larval stages inhabiting each pool, and were used to justify the inclusion of all open water bodies (except small anthropogenic containers such as bowls) in the Zindarou simulation.

Concurrent with the larval sampling, surface water body extents were regularly measured in the field. This was done with either a tape measure to determine the major and minor dimensions of each pool, or with a handheld GPS receiver for the larger pools with more complicated shapes. These dimensions were used as a validation for model-predicted pooling.

Many references cite very small depressions such as cattle hoof prints and tire ruts as primary anopheline breeding habitats [e.g. [11-13]]. In Banizoumbou and Zindarou, we encountered such micropools, but in all cases they have been directly beside larger, saturated topographic depressions and as such their existence is embodied in the model structure as lumped with larger pools. Cattle hoof prints and tire ruts were also seen at some distance from such depressions, however the persistence of pooled water in these microtopographic depressions is several hours at most, which is far too low to allow development of mosquitoes. Typical sizes of such micropools were several centimeters to tens of centimeters. Without exception the micropools not associated with larger pools were seen to disappear very rapidly after rainfall. With weekly, comprehensive surveys, we never observed late-stage larvae or pupae in cattle hoof prints or tire tracks, except when located at the saturated edges of larger pools. In Zindarou as in Banizoumbou, there is therefore no need to simulate at spatial scales consistent with such microdepressions.

**Figure 2:**
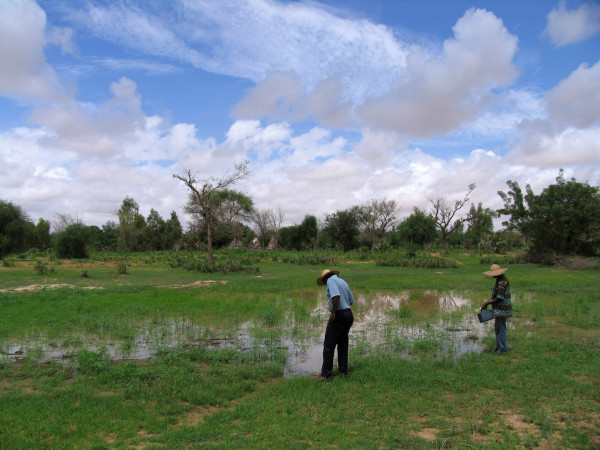
**An example of surface expression of groundwater in Zindarou, at a topographic low point**.

In Zindarou, the seasonal groundwater rise increases the extent of surface water availability for mosquito breeding in topographic low points. When the groundwater table is higher than the ground level of these topographic low points, large areas of open water form. An example is depicted in Figure 2. The presence of such extents of shallow open water is a fundamental difference in the hydrology of Zindarou compared to Banizoumbou. The water level fluctuations are incorporated into a version of HYDREMATS tailored to the Zindarou hydrological environment.

Villagers maintain their shallow hand-dug garden wells to water dry-season vegetable crops, and keep them deep enough to reach at least the groundwater table year-round. The garden wells are therefore permanent, however the large areas of surface groundwater expression are seasonal. One well has a concrete casing which provided a useful, permanent, non-intrusive measurement reference. During each field visit (once weekly), depth to water table was measured from this permanent reference datum to yield a time series of water table fluctuations at this location.

Groundwater level rise is also evident in some TDR soil moisture measurements. At two of the TDR measurement sites the lowest probe, at one meter depth, registered saturated conditions during the peaks of the wet 2005 and 2006 monsoons. The depth reference for each of the TDR soil moisture measurement points (sensors at 10 cm, 20 cm, 50 cm or 100 cm below the surface) was the ground surface just above the sensor. For each of the three sensor installations at Banizoumbou or Zindarou, these reference locations were surveyed using a survey-grade differential GPS system so that the elevations of each soil moisture sensor was known to centimeter precision. This allowed precise positioning of the sensors that became saturated. Because each sensor is sampled for soil moisture at regular intervals and the collected information stored in the datalogger, the exact time of the water table surface passing the known sensor elevation can be determined. From these measurements, a groundwater level time series was produced. The measurements show a very flat groundwater table around Zindarou, rising and falling as a flat surface seasonally, and the groundwater level is therefore assumed to be applicable over the entire 2.5 km square model domain surrounding Zindarou.

### Model structure and domain

We apply the model HYDREMATS to Banizoumbou and Zindarou. HYDREMATS is a coupled hydrology and entomology model that simulates in very high spatial and temporal resolution the formation and persistence of the types of pools that facilitate the breeding of *An. gambiae s.l*. mosquitoes [1]. Within HYDREMATS, water, energy, and momentum balances in two canopy layers and multiple soil layers are performed by a modified version of the Land-Surface Exchange model (LSX) of Pollard and Thompson [14]. For a particular model grid cell, this formulation simulates all relevant processes influencing the partitioning of incident rainfall into overland runoff flow, infiltration and surface evaporation. The procedure accounts for abstractions from vegetation root zone uptake, transpiration, evaporation and variable infiltration resulting from soil heterogeneities and antecedent moisture conditions, as well as changes in water storage for each model grid cell. Incident precipitation that is not abstracted in the soil column or in the vegetation is assigned to soil runoff for each grid cell.

An overland flow routing component was included in the model to route predicted runoff using a two-dimensional solution to the shallow flow equations as in [1]. Overland flow strongly depends on the land surface topography for both direction and velocity, and topographic low points represent basins that can impound runoff water to constitute mosquito breeding habitat. Spatially-distributed roughness from differing soil and vegetation surfaces, parameterized by Manning's *n*, retards overland flow. At each model grid cell and for each model time step, differences in runon from upstream cells and runoff to downstream cells results in a change in water depth, and the amount of water that runs off to downstream cells depends on the difference in water level between the adjacent cells but is affected by the roughness of a particular cell. The model steps forward through one-second time steps in this manner to simulate the land surface response to incident rainfall, and water is routed to the topographic low points over the infiltrating, evaporating surface. Once all runoff water from a storm has reached pools, simulated evaporation and infiltration continue to diminish the water contained in individual breeding habitats until either the pool dries out or it is replenished by overland flow from a subsequent precipitation event.

**Figure 3:**
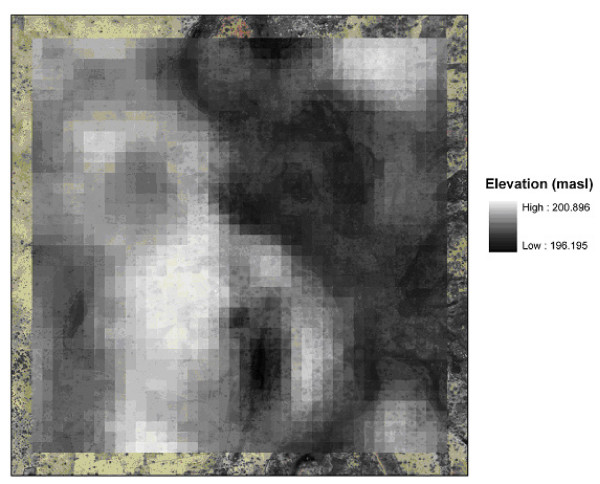
**Model domain, discretization, and topography for the 2 km × 2 km area surrounding Zindarou**. The model domain is superimposed on an Ikonos image of the Zindarou environs. Grid cells are 50 m square. Zindarou village is located at the center of this model domain.

The 2.5 km × 2.5 km model domain around Banizoumbou was chosen based on expected extent of mosquito dispersal around the village. For the same reason, as well as computational efficiency, a similar model domain was chosen surrounding slightly smaller Zindarou (2 km × 2 km). Figure 3 shows the model grid superimposed on an Ikonos satellite image of Zindarou. The village is located at the center of this figure, and the discretized model domain shows elevations applied to each model grid cell. Grid cell elevations are from a 50-meter resolution digital elevation model derived from Envisat satellite synthetic aperture radar data [15]. The model grid does not have the same very fine discretization as the center of the Banizoumbou grid (10 meters square), because in the center of the model domain near Zindarou, no rainwater pools of comparable size were noted. Only shallow garden wells and a few small saturated areas nearby large groundwater-induced surface water bodies were of this size. The garden wells were represented in the model as stationary, permanent small water bodies. Because they are smaller in size than the computational grid elements for Zindarou (50 meters square), unchanging water bodies of one meter square were superimposed on dynamic hydrology model output to represent their existence. In this way, unchanging surface water availability from garden wells was represented.

The entomology model development is discussed in detail in previous work [1]. However, details of the model relevant to this study are presented here. Hourly predictions of breeding pool locations and the pool water temperatures provide the input for the entomology component of HYDREMATS. These pools facilitate the breeding of Anopheles larvae and eventual eclosion as adult mosquitoes, provided the pool persists long enough for temperature-dependent development to complete. After emergence, simulated adult mosquitoes interact with their environment and disperse in the model domain in an agent-based model structure. Interactions with village inhabitants and water bodies for oviposition (egg-laying) allows mosquito population dynamics and malaria transmission to be tracked. After pools have received eggs from a gravid female, the predicted temperatures determine the pool-specific advancement rate of aquatic-stage development from egg to larva to pupa and finally adult mosquito [16]. From the integration of individual adult mosquitoes' actions, resulting population scale behaviour such as dispersal can be derived. For each mosquito agent, age, gonotrophic state, and number of blood meals are tracked in addition to its location. Only female mosquitoes are tracked, because male mosquitoes do not take bloodmeals and therefore play no role in malaria transmission. We assume that male availability for mating is not limiting. The full model parameterization is presented in Table 1[17-21].

**Table 1:** Parameterization for the entomology model (from [1])

**aquatic stage simulation**				
**Variable**	**nominal value**	**units**	**reference**	sensitivity*

cannibalism rate	0.0008	hr^-1^	[17]	medium
number of eggs lain per oviposition	150		[18]	low
egg death rate	0.001	hr^-1^	none	very low
weight of first-stage larvae	0.02	mg	none	low
weight of stage 2 larvae	0.16	mg	none	low
weight of stage 3 larvae	0.30	mg	none	low
weight of stage 4 larvae	0.45	mg	none	low
lag time for predators to establish	240	hr	[16]	low
carrying capacity of pools (at peak)	300	mg m^-2^	[16]	low
larvae death rate	0.005	hr^-1^	[19]	very low
maximum predation rate for larvae	0.006	hr^-1^	[16]	medium
pupae predation rate	0.005	hr^-1^	none	very low

**adult mosquito simulation and human individuals**				

**Variable**	**nominal value**	**units**	**reference**	**sensitivity**

human infection clearing rate	0.0005	hr^-1^	[19]	very low
degree days above 18°C necessary for sporozoites	111	deg-day	[20]	very low
average mosquito flight velocity	15	m hr^-1^	[6]	low
time required for gametocyte development	336	hr	[20]	very low
weighting of random walk vs. straight line	0.2		none	medium
resting time	24	hr	none	medium
threshold distance for visual cues	15	m	[21]	low
utilization probability of water	0.95		none	low

*parameter sensitivity gauged by maximum simulated abundance

Mosquito mortality is simulated according to the following equation [22]



where *p *is the daily survivability probability of each mosquito and *T*_*d *_is the average temperature of the previous 24 hours. Egg development within the mosquito follows the temperature-dependent model of Depinay et al. [16]. Ambient temperature at the mosquito's location regulates this development rate. If the simulated mosquito has finished the full gonotrophic (egg development) cycle, and it encounters a suitable simulated water body, then it deposits a clutch of eggs to add to the subadult mosquitoes of various stages already present in that water body.

In a particular pool, the total biomass from larvae is summed based on the numbers and weights of individuals. This total biomass is then compared to an ecological carrying capacity (a model parameter) to determine a coefficient of intra-specific competition which regulates the advancement of larval stages. Crowded conditions in the pools prevent larvae from getting enough nutrients and slow development and eventual pupation and eclosion [16].

### Adaptation of the entomology model to the Zindarou environment

While the decrease of Banizoumbou mosquito populations following cessation of rains coincides with the disappearance of primary breeding pools, the decline of the Zindarou anopheline populations did not coincide with the disappearance of surface water available for mosquito breeding. Large areas of surface water persisted into November as the groundwater table slowly receded, but the persistent pools did not produce large numbers of anophelines after early September. Clearly, other factors limited mosquito development and eclosion. Limitations may include competition for reduced nutrient sources such as pollen from nearby plants, and heavy predation after complex ecosystems have become established in the pools.

Predation can seriously affect mosquito populations, and constitutes a significant proportion of larval mortality [13]. Soon after the formation of pools following rainstorms, *An. gambiae *lays eggs which rapidly develop into adults. However, after this initial cohort of mosquitoes emerges, predators become established with some delay. This frequently results in a decline from initial population peaks as predators begin to regulate the population [13,16]. Predation's effect on larval abundance is represented in HYDREMATS as a logistic function. In this formulation, following pool formation predation rate increases with a sigmoid curve with a delay of several weeks to reach the maximum allowable predation rate. Both lag time and maximum predation rates are model parameters. In Banizoumbou, pools disappear much more frequently than in Zindarou, and predators have little time to become established before an individual pool dries. Rewetted pools reset the predation rates, and therefore the high predation rates of Zindarou are not seen in Banizoumbou.

Entomology model parameterization for Zindarou is the same as Banizoumbou, except for variable ecological carrying capacity in the Zindarou simulation. *An. Arabiensis *has been shown to feed on wind-bourne maize pollen in Ethiopia [23]. This abundant and nutritious food source increased productivity of breeding habitats that were nearby flowering maize, and much lower larval development was noted where maize pollen was absent [23]. No maize is grown near Banizoumbou and Zindarou, but the local staple crop pearl millet is nearly ubiquitous in the region. Similar to maize, pearl millet depends on wind-bourne pollen for reproduction [24]. It has not been shown that mosquito larval development is linked to millet pollen availability, but the large amounts of pollen produced by millet [25] and the wind-dispersal mechanism for reproduction, combined with the observed decline in Zindarou mosquito populations closely following the end of millet flowering suggest that the wind-dispersed millet pollen that lands on pool surfaces presents a nutritious food source to *An. gambiae *larvae. In the model, ecological carrying capacity is decreased from 300 mg m^-2 ^during the peak of the monsoon to 30 mg m^-2 ^after September 15^th ^to represent the end of the pollen availability. Observed declines in mosquito abundance cannot be reproduced without this decline in pool carrying capacity. The same reduction of carrying capacity in the Banizoumbou simulation would not affect the mosquito populations, because the Banizoumbou pools disappear due to lack of rain in mid-September, roughly at the same time as the reduction of Zindarou carrying capacity. Again, except for this difference all model parameters are identical for the two villages.

### Adaptations of the hydrology model to the Zindarou environment

**Figure 4:**
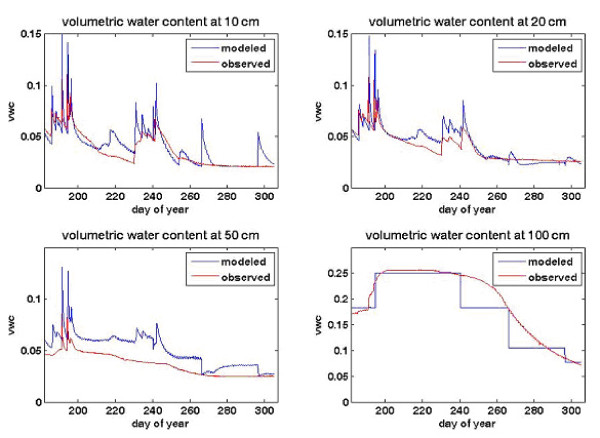
**Soil moisture simulated and observed values at the Zindarou South TDR recording site, for the 2005 rain season**. The influence of the groundwater table rise is evident in the 100 cm probe, as the volumetric water content reaches saturation for about six weeks. The modeled soil moisture reproduces the trend, but has a step shape because of the vertical soil discretization.

Distributed soil parameters are assigned based on previous studies performed in the area [e.g. [26]] as well as model calibration. Assigned soil parameters are summarized in Table 2. Example soil moisture model results are depicted in Figure 4 with each figure pane representing a different soil layer at the Zindarou South measurement site. Soil moisture measured by the TDR probes is compared to the model-predicted soil moisture. The 100-cm TDR probe shows the influence of the groundwater table rise, which is reproduced by the model. The stepped nature of the model output is due to the discretized vertical soil layers in the model.

**Table 2:** Zindarou hydrology model parameters (from [1])

	**Subsoil**		**crust**	
		
	**millet fields & fallow**	**garden area**	**millet & fallow**	**pool bottom**
saturated hydraulic conductivity K_s_	5.4 × 10^-2 ^mm sec^-1^	1.7 × 10^-2 ^mm sec^-1^	3.3 × 10^-4 ^mm sec^-1^	3.8 × 10^-4 ^mm sec^-1^
porosity θ_s_	0.25	0.44	0.38	0.48
Campbell's "b" exponent	1.3	1.85	6.0	7.6
Air entry potential Ψ_e_	-0.07 m	-0.08 m	-0.29 m	-0.37 m
Manning's n	N/A	N/A	0.12	0.05

The seasonally fluctuating water table in Zindarou is simulated using a modified version of the unsaturated zone hydrology model within LSX. The one-dimensional Richards equation governs vertical water redistribution, and Campbell's model [27] is used for soil water retention.

In HYDREMATS, the unsaturated zone model of LSX has been expanded to include representation of a groundwater table, following the formulation of Yeh and Eltahir [28] for simulations in shallow groundwater aquifers in Illinois. At the boundary of the saturated and unsaturated zones, the difference of gravity and diffusion fluxes determines net recharge into (or out of) the aquifer for each time step. For each time step, the aggregate net recharge integrated over the model domain determines water level change and hence updated groundwater level for the subsequent time step. In this manner, water table fluctuations are simulated. Air entry potential *ψ*_e _and Campbell's *b *exponent were used as calibration parameters for groundwater level.

The formulation of Yeh and Eltahir [28] was modified to allow the groundwater table to penetrate the surface and allow pooling. This involved coupling with the overland flow routine to combine surface water bodies formed by groundwater table rise with pooled water from overland flow. The diffusion wave formulation of overland flow routing represents backwater effects, allowing multicell pooled areas to form when overland flow meets surface-penetrating groundwater.

Necessary model input data included topography, vegetation and soil types. These were assigned by supervised classification of a Landsat 7 multispectral image with ground resolution of 30 meters [15]. For model grid cells larger than this spatial resolution, the land cover class represented by the most are from the image was assigned to the model grid cell. Field investigations of soil type (90% sand) and detailed verification of vegetation informed the model as well. Topography was surveyed in April 2008 using a highly accurate differential GPS system, at very fine spatial resolution that allowed a 10-meter digital elevation model to be generated.

## Results

### Hydrology

**Figure 5:**
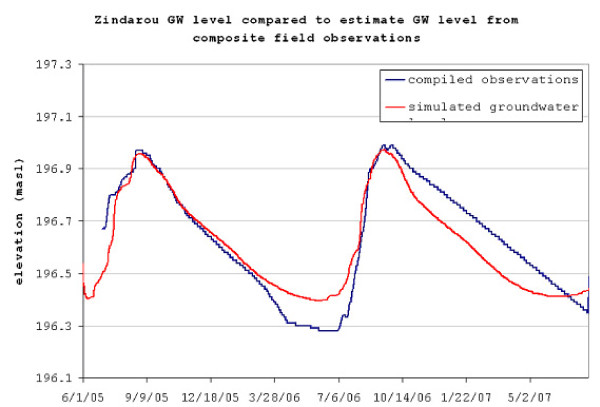
**Comparison of observed and simulated groundwater levels in Zindarou**. Near the wet season groundwater peaks, the observed groundwater level contains many measurements, whereas during the dry season measurements are sparse.

Figure 5 compares results of the groundwater simulation to the observed water levels. Discrete measurements of water levels at groundwater wells, observations of saturation and subsequent dry down at buried TDR probes, and observations of extents of groundwater-fed pools were compiled into a combined representation of groundwater level. Observations were at least weekly at all sites during the wet seasons, but little data was available for defining domain-wide groundwater behaviour during the ensuing dry season. Only one concrete-lined well provided periodic measurements of depth to groundwater table. Buried TDR probes registered the dry-down, and slow dessication of the large swampy areas around Zindarou in November and December provided clues about the extent of groundwater table recession during the dry season. However, compared to the wet season when many areas showing surface penetration of groundwater existed and were measured, observations of groundwater behaviour in the dry season are sparse. The simulated groundwater table fluctuations are superimposed on the observed groundwater level, showing good agreement in the 2005 rain season and less good agreement during the dry season. However, due to the deficiency of reliable measurements throughout the region as well as the inconsequence of groundwater levels when there are no areas of surface penetration, divergence of simulated groundwater level from observations is considered acceptable during the dry season. Peak groundwater levels match observations well, and due to relatively shallow topography, pooled water surface area is sensitive to maximum rise of the groundwater table. Therefore, the accurate model reproductions of Zindarou groundwater levels shown in Figure 5 signal good model representation of surface water availability for mosquito breeding.

**Figure 6:**
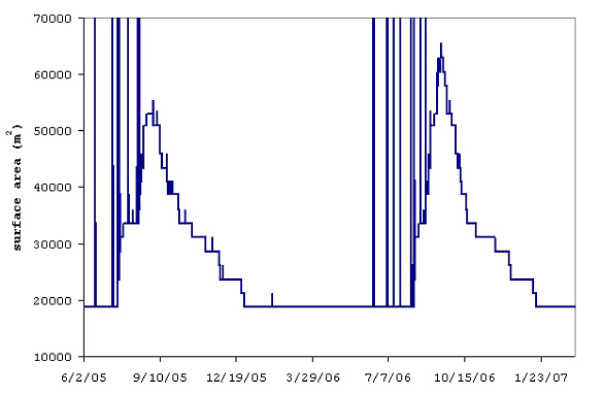
**Simulated surface area of water (m^2^) over the entire Zindarou model domain**. There are many areas with year-round surface water, including wells, ditches and swamps.

Figure 6 shows surface water area in the model domain over the simulation period. Total water surface area summed from various sources (groundwater-saturated zones, shallow garden wells, and overland flow) is represented in this plot. Water surface area from overland flow (often submerging much of the model domain for one time step as water is being routed to the topographic low points) disappears rapidly compared to groundwater-supplied marshes, which provide the longer-duration surface area.

### Mosquito abundance

**Figure 7:**
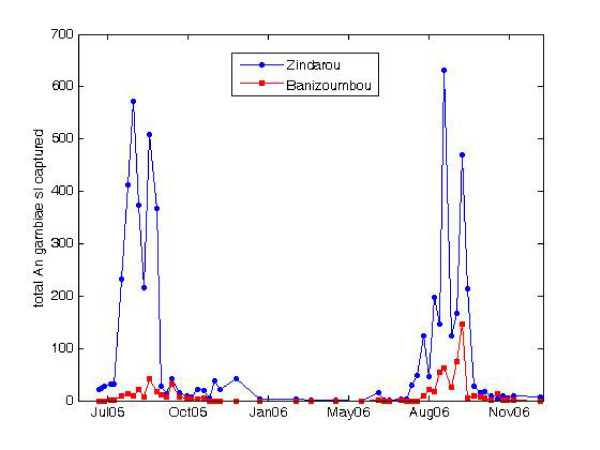
**Total captured *Anopheles gambiae s.l*. mosquitoes captured in Banizoumbou and Zindarou in the period June 2005 - December 2006**.

Total mosquito captures in Zindarou and Banizoumbou are shown in Figure 7. In Banizoumbou, light traps yielded only *An. gambiae s.l*. mosquitoes (the species complex which comprises sibling species *An. gambiae sensu stricto *and *An. arabiensis*). In Zindarou, light traps also showed *An. gambiae s.l*. to be the most abundant species. Due to resource limitations, the sibling species *An. gambiae s.s*. were not distinguished from *An. arabiensis*, because they are morphologically identical and require genetic analysis for identification beyond the species complex level. *An. funestus *were also caught albeit in much lower numbers. Due to its anthropophily, *An. funestus *is an efficient malaria vector [29]. Other captured anophelines included *An. rufipes *and *An. pharoensis*, however total captures for these species were very low even in Zindarou, and because of lower anthropophily they are considered only minor vectors of malaria [30]. We therefore assume that they play a negligible role in Zindarou malaria transmission and omit them from the study.

**Figure 8:**
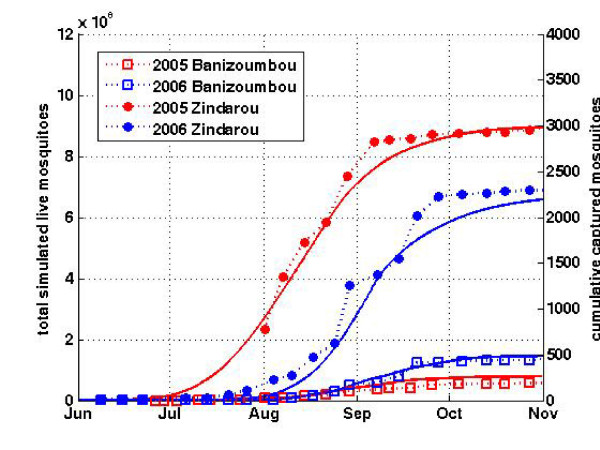
**Cumulative simulated and observed mosquitoes in Banizoumbou and Zindarou**. Close fits of simulation results to observations show that the model reproduces the inter-village differences in mosquito abundance as well as the interannual variability in abundance between the villages.

Weekly light trap catches at six locations throughout the village provided the field observations for mosquito abundance. Differences in ambient light such as from lunar phase are known to influence light trap captures [31,32], and overnight captures are a time-integrated sampling method. Total time-integrated mosquito abundances, both from light traps and from model predictions in each village, compare well. Because light traps effectively represent relative abundances, the interannual difference in simulated mosquitoes can be compared to the interannual difference in observed (captured) mosquitoes, assuming that the light trap samples a constant proportion of the active mosquitoes in the village. Figure 8 shows 2005 and 2006 simulated cumulative and observed mosquito abundance in Zindarou compared to simulated cumulative and observed mosquito abundance in Banizoumbou for the same two years.

Despite successful representation of hydrology-driven mosquito population variability between the two villages, the modeling approach has limitations. The simulation is highly detailed, but it was not possible to validate the model at the hourly time step of simulation, nor was it possible to compare the details of modeled intra-village population structure beyond the light trap locations. Model output at very small spatial- and time-scales (not presented) cannot be completely validated and while model output is realistic at such scales, details should be interpreted with caution. Emphasis is placed on the representation of intervillage difference in time-integrated mosquito abundance to highlight the effects of hydrological variability.

## Discussion

Banizoumbou and Zindarou Villages are only 30 km apart, but are hydrologically very distinct. Besides the contrast in surface water availability for breeding mosquitoes, the differences between the villages are minimal. It is therefore expected (and observed) that potential confounding variables such as climate or vector behaviour do not vary significantly between the two villages. With highly detailed representation of localized hydrological determinants, HYDREMATS has reproduced both the interannual variability in mosquito abundance observed in the two villages and the pronounced differences in abundance observed between Banizoumbou and Zindarou. The only differences in the simulations were the local topography, distributed land cover type, and subsurface hydrology environment as observed for each study village. These heterogeneities led to pronounced inter-village differences in breeding habitat formation and persistence, and ultimately result in highly variable village malaria risk. In the comparison of Banizoumbou and Zindarou, subsurface hydrology was seen to be the dominant control of inter-village difference in entomological activity. The influence of the Dallol Bosso and the associated shallow groundwater table resulted in prolonged and widespread breeding habitat availability.

The implications of the observations and model results presented in this study are important for accurate and precise mapping of malaria risk in water-limited, epidemic-prone African environments. The wet environment of Zindarou with persistent surface water resembles hydrological conditions typical of hundreds of kilometers further south, in the wet equatorial forests of West Africa, however temperatures and humidity are characteristic of the more arid Sahel. Clearly, malaria risk as indicated by mosquito abundance is higher in Zindarou-like environments than in more dry Banizoumbou-like environments. While Zindarou represents one end of a spectrum of wetness and corresponding entomological activity in the Sahel, mosquito abundance in many villages in the Sahel will fall somewhere in between that of Zindarou and Banizoumbou. As previously stated, hydrological variability is low at the dry northern and wet southern fringes of the Sahel and Sudan Savannah zones, and inter-village variability in malaria risk due to variable mosquito activity is expected to follow this trend.

Wide-ranging characterization of such highly detailed spatial data to parameterize a distributed model for topography, soil, vegetation and climate data can be a formidable challenge. The results presented in this study involved supervised classifications of multispectral satellite images for model input, and intensive field investigation for the characterization of the surface expression of groundwater in Zindarou. In some cases, ground truth of satellite information for land cover can be extrapolated regionally to nearby villages in the Niger Sahel with similar soil and vegetation types for unsupervised classifications, with reasonable assurance of validity.

Results of this study can have significant implications on the downscaling of climate model output to the regional scales for making malaria transmission projections. Similarly, the presented results also have implications on the development and application malaria early warning systems (MEWS). Climate models and MEWS often operate at low spatial and temporal resolution, typically on the order of hundreds of kilometers. MEWS systems use the detection of anomalous conditions in sea surface temperature, seasonal climate forecasts as well as regional vulnerability assessments based on the human population to predict increased malaria transmission, with varying degrees of success. The impact of uniformly-distributed precipitation anomalies on village-scale malaria transmission depends on the hydrological characteristics of each individual village. Generally, within MEWS, positive precipitation anomalies are expected to yield regionally intensified malaria transmission. However, as with the difference between Banizoumbou and Zindarou, the details of the malaria transmission response to rainfall anomalies will vary with localized hydrological determinants. Following results of this study, a large-scale prediction of epidemic conditions from an early warning system should be refined using an application of detailed simulations such as HYDREMATS to increase specificity and sensitivity of the system, identifying populations truly at risk while negating false warnings.

While pool persistence limits mosquito abundance in Banizoumbou, in wetter environments such as represented by Zindarou, different modes of limitation regulate abundance particularly in the late wet season. Nutrient availability and predation may be significant controls in the late transmission season, as is evidenced by a drastic decline in late season observed mosquito populations despite the persistence of groundwater-fed pools beyond the end of the rains. The difference in pool persistence resulting from spatial hydrologic variability will influence local modes of mosquito population limitations.

This field-validated modeling study of Banizoumbou and Zindarou, Niger, has exposed the importance of high resolution hydrological simulation for improved understanding and simulation of village-scale malaria transmission. Pronounced differences in observed mosquito populations between the villages were reproduced by the HYDREMATS model, demonstrating the potential for improved simulation of malaria risk.

## Conclusion

Spatial hydrologic variability controls local, village-scale mosquito abundance in areas of water-limited, seasonal malaria transmission such as the Niger Sahel. The example of Banizoumbou and Zindarou in this study showed that a distance of only 30 km between the two study foci can have dramatically different anopheline abundance, and that these differences were solely explained by hydrologic variability. This result has implications for efforts to predict malaria risk using climate models. The mismatch of climate model grid cell size (typically ~100 km) and the characteristic scale of hydrological variability that influences village-scale malaria transmission in areas such as the Sahel suggests that malaria risk predicted by climate models (as components of early warning systems) may contain limitations. Accurate and reliable assignment of village-scale malaria risk from such models may be impossible because of this scale discrepancy. Moreover, the result suggests that high resolution distributed representation of hydrology may dramatically improve the model-predicted outcome of malaria transmission response to variable climate forcings. Topography, vegetation, soil type differences as well as shallow groundwater behaviour must all be incorporated at appropriate scales in order to accurately evaluate malaria transmission at the village scale using coarse resolution climate models.

## Competing interests

The authors declare that they have no competing interests.

## Authors' contributions

AB conducted the field data collection, modeling study and drafted the manuscript. JBD provided guidance and field expertise in anopheles mosquito ecology and field methods. EE conceived of the study. All authors have read and approve of the final manuscript.
